# The Correlation of Online Health Information–Seeking Experience With Health-Related Quality of Life: Cross-Sectional Study Among Non–English-Speaking Female Students in a Religious Community

**DOI:** 10.2196/23854

**Published:** 2020-12-02

**Authors:** Zahra Kavosi, Sara Vahedian, Razieh Montazeralfaraj, Arefeh Dehghani Tafti, Mohammad Amin Bahrami

**Affiliations:** 1 Health Human Resources Research Center, Department of Health Services Management School of Management and Medical Informatics Shiraz University of Medical Sciences Shiraz Iran; 2 Healthcare Management Department Shahid Sadoughi University of Medical Sciences Yazd Iran; 3 Department of Biostatistics and Epidemiology Kerman University of Medical Sciences Kerman Iran

**Keywords:** general health, SF-36, information seeking behavior, online health information, high school students, health literacy

## Abstract

**Background:**

Given the increasing availability of the internet, it has become a common source of health information. However, the effect of this increased access on health needs to be further studied.

**Objective:**

This study aimed to investigate the correlation between online health information–seeking behavior and general health dimensions in a sample of high school students in Iran.

**Methods:**

A cross-sectional study was conducted in 2019. A total of 295 female students participated in the study. The data were collected using two validated questionnaires: the e-Health Impact Questionnaire and the 36-Item Short Form Health Survey. The collected data were analyzed through descriptive statistics and Pearson correlation coefficients using SPSS version 23 (IBM Corp).

**Results:**

The participants moderately used online information in their health-related decisions, and they thought that the internet helped people in health-related decision making. They also thought that the internet could be used to share health experiences with others. Participants had moderate confidence in online health information and stated that the information provided by health websites was moderately understandable and reliable and moderately encouraged and motivated them to play an active role in their health promotion. Nevertheless, the results showed that online health information–seeking experience had no significant correlation with health-related quality of life.

**Conclusions:**

This study provides insights into the effect of using internet information on the health of adolescents. It has important implications for researchers and policy makers to build appropriate policies to maximize the benefit of internet access for health.

## Introduction

Adolescence refers to the age range of 10 to 19 years [1]. It is generally supposed that this period is an appropriate time to maintain and promote health and prevent health-related adverse effects in the following decades of life [2]. Despite this potential, adolescents have special needs that are often not well met by health systems [3]. Evidence suggests that many high-risk behaviors that usually begin in adolescence cause an epidemic of noncommunicable diseases in adulthood [4]; an 18-year prospective study has shown that physical activity in adolescence has a significant effect on one’s health in adulthood [5]. Today, adolescents are facing multiple health-threatening factors, various questions on different aspects of health, and more complicated health challenges and problems than their parents did [6]. Studies on adolescent health status highlight the necessity of changing the assumption that adolescents are generally healthy and need less attention [7]. Therefore, the question is where adolescents can receive help or information when faced with such challenges. Family, peers, teachers, health specialists, and online resources are common sources from which adolescents seek information and advice on health challenges [8].

In general, people choose different ways to find answers to their questions and doubts about health. Health information–seeking behavior refers to seeking and receiving information to reduce uncertainty and doubts and ensure health status [9]. As Wilson suggests in his model, “information-seeking behaviour arises as a consequence of a need perceived by an information user, who, in order to satisfy that need, makes demands upon formal or informal information sources or services, which result in success or failure to find relevant information” [10].

Similar to most fields, health information seeking has changed from traditional practices such as referring to books and magazines and even direct expert advice to new methods such as the use of the internet and social networks. Online resources play an important role in providing health information, and young people are increasingly using online information in various domains. In their systematic review, Park and Kwon (2018) showed that adolescents used the internet widely in different countries [11].

According to another systematic review, 81% (21/26) of the studies indicated that more than 50% of their samples used the internet to obtain health information [12]. Studies show that adolescents use the internet to find answers to their wide range of health-related questions; on the other hand, they doubt the comprehensibility and validity of online information [12-14].

Johnson et al (2015) found that youth with lower mental quality of life used the internet more to gain health information [14]. Besides, studies have shown that adolescents with more health risk factors and those with worse health status, higher health literacy, and a chronic disease are more likely to use the internet to search for health information [15]. In this regard, a question that has remained as a main concern is whether adolescents have sufficient ability to effectively search for, evaluate, and use online health information in a way that promotes their health [16,17]. Thus, the adolescents’ ability to access health information online can be described as a double-edged sword that may have a positive or negative impact on their health.

According to the 2016 census in Iran, adolescents make up 8% of the country’s population of 12 million, half of whom are girls [18]. Iran has one of the highest rates of internet access in its region [19]. Since a high percentage of the Iranian population is composed of adolescents and youths, and due to the cultural and religious contexts of the country, some of the challenges that adolescents face are not disclosed to their parents or professionals. Therefore, the internet seems to provide an opportunity through which they can seek answers to their health-related questions. Hence, this study aimed to investigate the relationship between online health information–seeking behavior and general health status on a sample of high school girls in Iran. We were particularly interested in studying the online health information–seeking behavior and its correlations with health outcomes among female students for several reasons. First, according to statistics from the Ministry of Education, girls make up half of all Iranian students [20]. Second, adolescence is a critical period of life regarding health, especially for health-promoting behaviors. Statistics show that one fifth of the world’s population is between the ages of 10 and 19 years, and 85% of them live in developing countries. Promoting adolescents’ health is one of the national development goals, and satisfying the health needs of this population is among the top priorities of health systems around the world. Changing adolescents’ health-related behaviors and their lifestyles requires providing them with appropriate and complete health information [21]. Third, girls play an important role in the health of today’s and future society, and investment in improving their health is one of the most important strategies to achieve global health goals [18]. The fourth reason is that there is a growing body of research that explores the significance of context in health information, demonstrating that gender is a determinant of information-seeking behavior. Many authors agree that health information seeking is influenced by gender [22]. In a study by Rowley et al (2016), they confirmed gender as a factor influencing the process of health information seeking and evaluation [23]. In addition, some other studies have reported important gender differences in health information–seeking behavior [22-26]. Therefore, it is crucial for societies to help female students to maintain and promote their health, which was the aim of this study as well.

## Methods

### Overview

This cross-sectional questionnaire study was conducted in 2019. A total of 295 female high school students in Ardekan city, Yazd province, who had access to the internet and the experience of health information seeking participated in the study. All participants provided informed consent to participate in the study and were assured that their personal information would be kept confidential. The parents of the students were made aware of the participation of their children in the study and had the opportunity to not let their children participate in the study. The school principal and students’ teachers approved the study. All the study procedures were conducted in accordance with the ethical standards of the Declaration of Helsinki. In addition, the ethics committee of Shahid Sadoughi University of Medical Sciences approved the study (approval code: IR.SSU.SPH.REC.1399.023). Questionnaires were completed in class, and any students who were absent on the testing days had the opportunity to participate in the study on the following days. All the data were gathered using two validated questionnaires: the e-Health Impact Questionnaire (eHIQ) and the 36-Item Short Form Health Survey (SF-36).

### eHIQ

The eHIQ was used to measure the online health information–seeking behavior of participants. The eHIQ, developed by Kelly et al in 2015 as an instrument to measure the potential consequences of using websites containing different types of material across a range of health conditions, is a 2-part instrument with 37 items. eHIQ-Part 1 consists of 11 items related to general views of using the internet in relation to health. These 11 items have been grouped into 2 subscales named “Attitudes towards online health information” (5 items) and “Attitudes towards sharing health experiences online” (6 items). eHIQ-Part 2 consists of 26 items related to the consequences of using specific health-related online sources. The 26 items have also been grouped into 3 subscales: “Confidence and identification” (9 items), “Information and presentation” (8 items), and “Understanding and motivation” (9 items). In our study, the participants were asked to respond to the 26 items of eHIQ-Part 2 regarding the online sources from which they have sought information in recent months. In addition, the participants were asked to score all items from both parts on a 5-point scale ranging from 1 (“never”) to 5 (“always”). We used a standard “forward-backward” procedure to translate the eHIQ from English into Persian. To demonstrate the content validity, we used the content validity ratio to quantify the extent of the experts’ agreement. The reliability of the translated version of the eHIQ was confirmed using the Cronbach alpha coefficient, which was calculated as 0.89 for the total scale and 0.81, 0.87, 0.94, 0.83, and 0.91 for “Attitudes towards online health information,” “Attitudes towards sharing health experiences online,” “Confidence and identification,” “Information and presentation,” and “Understanding and motivation,” respectively.

### SF-36

The SF-36 is a popular instrument for assessing the health-related quality of life. The SF-36 has 36 items, which measure 8 subscales (ie, vitality, physical functioning, bodily pain, general health perceptions, physical role functioning, emotional role functioning, social role functioning, and mental health). These 8 subscales of SF-36 are grouped into two distinct dimensions, namely a physical dimension represented by the physical component summary (PCS), which is the sum of physical functioning, bodily pain, general health perceptions, and physical role functioning, and a mental dimension represented by the mental component summary (MCS), which is the sum of vitality, emotional role functioning, social role functioning, and mental health. After completing the questionnaire, each scale is directly transformed into a 0-100 score on the assumption that each question carries equal weight. The lower the score, the greater the disability; the higher the score, the less the disability (ie, a score of 0 is equivalent to maximum disability and a score of 100 is equivalent to no disability). In this study, we used the Persian version of the SF-36, which had been validated by Montazeri et al (2005) [27]. In addition, we used the original scoring system. The collected data were analyzed through descriptive statistics (including means and standard deviations) and Pearson correlation coefficients, using SPSS version 23 (IBM Corp).

## Results

Of the participants, 16 students were married, and the rest were single. All of them had access to the internet at their home and the experience of seeking health information in recent months before the study. Demographic characteristics of the participants are presented in Table 1.

**Table 1:** Demographic characteristics of the participants (N=295).

Variable			n (%)
**Marital status**			
	Single		279 (94.6)
	Married		16 (5.4)
**Religion**			
	Muslim		279 (94.6)
	Not available		16 (5.4)
**Education level of parents**			
	**High school**		
		Fathers	104 (35.3)
		Mothers	116 (39.3)
	**Diploma and associate degree**		
		Fathers	122 (41.4)
		Mothers	108 (36.6)
	**Bachelor and higher**		
		Fathers	69 (23.4)
		Mothers	71 (24.1)

The findings regarding information-seeking behavior of the participants are presented in Table 2, showing that the participants have moderate scores on all subscales of eHIQ-Part 1 and Part 2. In this study, mean scores between 1 and 2.33, between 2.34 and 3.66, and higher than 3.66 were defined as low, moderate, and high levels, respectively. The moderate scores obtained by the participants in the 2 subscales of eHIQ-Part 1 indicated that the participants had used the internet moderately in their health-related decisions and thought that internet could be moderately useful to help people in their health-related decision making. They also thought that internet was a moderately good channel to share the health experiences and communicate with some people with the same health problems. In addition, the moderate score of participants regarding confidence and identification revealed that they did not have a sense of solidarity with other internet users in their information-seeking journey; the internet did not give them a sense of confidence to explain their health issues to others, and they thought that online searching did not help them to better manage their health-related conditions. Therefore, they did not highly value the online health information. The moderate scores of the participants regarding the last 2 subscales of eHIQ-Part 2, “Information and presentation” and “Understanding and motivation,” showed that the information provided by health websites had been moderately understandable and reliable for the participants and moderately encouraged and motivated them to play an active role in their health promotion.

**Table 2:** Mean scores for online health information–seeking behavior of the students.

Item		Mean score (SD)
**eHIQ-Part 1**		
	Attitudes towards online health information	2.46 (0.80)
	Attitudes towards sharing health experiences online	2.77 (0.90)
**eHIQ-Part 2**		
	Confidence and identification	2.52 (0.77)
	Information and presentation	2.90 (0.79)
	Understanding and motivation	2.90 (0.88)
eHIQ (total)		2.71 (0.71)

The descriptive results regarding the students' health statuses on the SF-36 subscales are presented in Table 3. As shown in this table, the participants had moderate to good scores on the SF-36 subscales. They obtained the highest and lowest scores in physical functioning and emotional role functioning, respectively.

**Table 3:** SF-36 scores of the students.

Item	Mean score (SD)
Physical functioning	83.67 (15.00)
Physical role functioning	75.94 (26.65)
Bodily pain	71.84 (23.27)
General health perception	63.31 (19.53)
Emotional role functioning	56.01 (38.58)
Vitality	75.94 (26.65)
Social role functioning	70.25 (25.34)
Mental health	65.29 (22.54)
Physical component summary	72.90 (16.20)
Mental component summary	63.19 (22.26)

The correlation coefficients of online health information–seeking behavior and its subscales with the main SF-36 subscales are presented in Table 4. Based on the findings presented in this table, eHIQ and its subscales showed no statistical correlation with SF-36 subscales. These findings suggest that seeking health information through online sources does not improve health-related quality of life. This could have several explanations. In the Discussion section, these explanations are discussed and suggestions are provided.

**Table 4:** Correlations of online health information–seeking subscales with health status.

Item	PCS		MCS	
	r	*P* value	r	*P* value
Attitudes towards online health information	0.04	.51	0.04	.55
Attitudes towards sharing health experiences online	0.05	.42	0.04	.50
Confidence and identification	0.02	.69	0.02	.67
Information and presentation	0.05	.38	0.05	.41
Understanding and motivation	0.03	.65	0.01	.84
eHIQ (total)	0.04	.46	0.04	.53

## Discussion

### Principal Findings

This study aimed to examine the correlation of online health information–seeking behavior with health-related quality of life in a sample of Iranian female students. Results showed that the participants used online information moderately in their health-related decisions and thought that the internet helped people in health-related decision making and could be used to share health experiences with others. Participants had a moderate amount of confidence in online health information. They stated that the information provided by health websites was moderately understandable and reliable, and it moderately encouraged them to play an active role in their health promotion.

Use of the internet to access health information has increased in recent years for reasons such as accessibility, high volume of information disseminated, confidentiality, low cost, multimedia capabilities, and the ability to interact and gain support [19,21,28]. Reports indicate that adolescents are increasingly spending their time on using the internet. Using the internet is part of young people's daily activities, and they acquire and enhance many life skills, including health management, through online information [28].

A US national survey has found that 75.0% (907/1209) of online teens search health information [29]. A study in the United States has also reported that 98.0% (200/204) of youth 12 years and older use online resources to search for health information [30]. Another survey at two US educational institutes [31], a study at three Ghanaian universities [28], a study involving international students in East Asia [32], and a study at six colleges in Oman have reported similar results [33]. Therefore, although internet access is still limited in some countries [34,35], it seems that the internet is increasingly becoming one of the main information sources in the majority of countries.

In Iran, as in other countries, using the internet for health-related purposes has increased in recent years. A survey of adolescents in Shiraz, Iran, has shown that the internet is among the top sources of the respondents' health information, with 88% (352/400) using the internet to find a kind of health information [36]. Two other studies in Tehran high schools have reported similar rates [37,38]. Another study on students aged 15-18 years from different schools in Isfahan [21], a study involving 430 students from Gonabad University [39], two other studies at Gorgan and Kermanshah universities [40,41], and two other studies at Tabriz University and Tehran University of Medical Sciences have reported similar results [19,42].

Overall, it seems that use of the internet as a source of health information is expanding; however, the review of the literature shows that searching for online health information is correlated with some variables such as age, gender, education level, skills and experience with internet use, health status, and availability and reliability of sources [1,31,43].

Adolescents often seek health information with different objectives and motives [36,40,42], and they typically seek information related to a variety of health subjects such as healthy eating, physical activity, exercise, weight control, risks and complications of disease treatments, sexual and reproductive health, sexual and physical abuse, consumption of alcohol and other substances, tobacco use, mental health, accidents and injuries, health care providers, and support groups [21,29,31,34,42].

Due to the increasing use of the internet for health purposes, many studies have been conducted on online health information–seeking behavior in different demographic groups, including students. Most of these studies have examined the sources of health information used by different groups, attitudes towards health information seeking, aims and motivations, types of information sought, and factors related to health information–seeking behavior [36]. However, few studies have examined the actual effect of accessing online health information on health status. In fact, the question of whether online health information–seeking behavior significantly affects health status or not has largely remained unanswered. Therefore, this study aimed to explore the online health experience of Iranian female students and its correlation with their health-related quality of life.

The findings showed that the majority of the participants had good or somewhat good general health status. Numerous studies have been conducted on the general health status of adolescents in Iran; most of them have reported approximately similar findings [18].

In addition, the descriptive findings of the study regarding online health information–seeking behavior showed that the participants had moderate scores on all subscales of eHIQ.

Regarding attitudes about online health information and sharing them, a similar study that aimed at explaining health information behavior of adolescents in Shiraz has reported that the participants’ general attitude toward health information retrieved from the internet is positive. The majority of the participants also trusted in the quality of information and were interested in retrieving health information from the internet twice [36]. Another study at Tabriz University has reported that the internet is considered one of the trustable sources of health information by participants [42]. At the same time, a study in Isfahan schools has shown that 47.7% (3110/6519) of those who did not use the internet to search for health information reported a lack of trust in the internet information as the main cause of their decision not to be an online health information seeker [21]. Regarding the sharing of health information, a study in United States has found that although 98.0% (200/204) of the participants were online health information seekers, only 51.5% (105) of them shared their health information and only 25% (51) of them thought that social media could provide usable health information. This study also reported that women had shared their health information more than men, and adolescents between the ages of 12 and 14 years had shared more than other age groups. People with poor self-reported health and those who thought online sources could help them in accessing health information were also more likely to share their health information [30]. Another study, which was conducted in India, reported that most of its respondents shared online health information with their friends and family [44]. In summary, based on the available literature, it seems that trust in online health information and interest in sharing it are different across different socioeconomic contexts. The participants of our study also thought that information provided by health websites was moderately understandable. In this regard, many studies have reported poor understandability of internet information as one of the main challenges for online users.

This study was conducted among a non–English-speaking female sample in a developing religious community. The unique features of the research environment may affect the results. Several studies show that contextual factors may affect different aspects of information-seeking behavior. Dankasa (2017) found in a study that geographical location, culture, and religious status may influence the information-seeking behavior of the internet users [45]. Lee and Cho (2011) and Chang and Lee (2001) have also reported the same results [46,47]. Based on the findings of these studies, contextual factors may encourage, determine, or prevent information-seeking behavior [45]. In addition, Lee and Cho (2011) found that social and cultural affiliations of individuals influence the way they choose to exchange information [46]. Therefore, our findings regarding the attitude toward online information and attitude toward sharing the information could be affected by the specific context of the study. Furthermore, this study was conducted among a sample of female students. Various studies have demonstrated that demographic variables such as gender and age, together with other factors such as income and education level, may influence health information behavior. Among these factors, gender has been widely identified as a factor affecting health information behavior. Accordingly, most studies suggest that being female and younger is associated with more frequent health-related use of the internet, although a few studies have reported contradictory findings [23,25,26,48]. The findings of this study can also be discussed based on the participants’ native language. Few studies have investigated information-seeking behavior of non–English language speakers or information-seeking behavior using non-native language. Although an increasing number of databases have now been created and made available in other languages, including Persian, English is still the dominant language of online information. Searching in different languages might affect different aspects of information-seeking behavior such as understanding of retrieved information, interpretation, evaluation, and the relevant judgment [49]. In this regard, some studies have reported differences between information seeking in different languages [49], while some have not confirmed the same differences [50]. There is no doubt that the users' language skills can affect their information-seeking behavior. In this study, it seems that all the studied subscales of information-seeking behavior include attitude toward online information, attitude toward sharing of information, confidence and trust, attitude toward the presentation, and understanding of information. It is notable that many studies have identified that the users’ attitude has a positive effect on their health information–seeking behavior, similar to the trust they placed in the information [23,26]. Therefore, it should be a priority to improve the attitudes of our participants toward their confidence in online health information.

Statistical tests also showed that different dimensions of online health information–seeking behavior had no significant correlations with health-related quality of life. On this subject, in a survey of 400 school-age adolescents in Shiraz, respondents stated that they believed that the retrieved online health information affected their health status positively [36]. In another study at Tabriz University, the participants approved of the effects of their online health information seeking on some health-related behaviors [43]. A study among Nigerian students found that only 50% (20/400) of participants consulted with a physician about their health after searching online health information [34]. A study at three universities in Ghana also reported that 72.4% (315/435) of respondents used retrieved online information as a basis for lifestyle modifications, and 73.6% (320) of the students stated that access to online health information improved or partially improved their health status, while 1.1% (5) said that using the internet had no effect on their overall health [28].

Overall, it seems that although internet technology has provided a good opportunity to access health information, its practical impact on health status is still controversial. This can have many explanations. Challenges such as the lack of appropriate information, inadequate quality of information, poor health literacy of internet users, insufficient skills in searching for information, lack of trust in online health information sources, and concerns about security and confidentiality reduce the potential of the internet in serving the health of population [21,29,42]. The production and dissemination of health misinformation is also a serious concern. Today, a great deal of health misinformation is also produced and published online, which is potentially a threat to public health [51]. Low internet access is also an infrastructure challenge in some parts of the world [34]. Therefore, it is necessary to formulate and apply improvement strategies to maximize the health benefits of internet. These strategies can be formulated in two levels: supply-side strategies (eg, expanding internet access; providing high-quality, appropriate, and understandable information; monitoring online health content; engaging health professionals in producing evidence-based information; ensuring safety; paying attention to legal issues; and focusing on adolescent health priorities [21,28,34,36]) and demand-side strategies (eg, investigating the patterns of use, improving health literacy, training search and information validation skills, and enhancing information behavior [29,34,36,40]).

Based on the findings of this study, interventions such as encouraging students to make more use of the internet as a source of health information; expanding their access to reliable online health sources; launching specific students’ health websites containing relevant, reliable, and understandable information by health authorities, especially in native language; improving the English language skills of students (since it could be a barrier for most of the participants in searching activities); improving students’ internet skills; and familiarizing them with search methods and specialized sources can be prioritized in order to maximize the potential of use of the internet in promoting the students’ health. It is also helpful to strengthen the online culture by using social marketing in the school environment. This study has several strengths. Few studies have been conducted in Iran to investigate the correlations between online health information–seeking behavior and the health status of students. In addition, there are few studies investigating the health information–seeking behavior of Persian language speakers. Therefore, the study has implications for research and practice. It contributes to research on health information–seeking behavior as it brings out the association of health information seeking with health outcomes that has not been given much attention in the literature. In addition, the study provides health and information professionals with information needed to make health information understandable, available, and accessible for students. The findings could also be used to develop appropriate interventions to enhance the students’ internet skills, so that they can make the best use of internet technology to promote their health. The study, however, has some limitations; first, it used a sample of female students, while some studies have reported gender-based differences in health information–seeking behavior that may affect the generalization of our findings to other population groups. Also, the study was done in a specific geographical, cultural, and religious context, which also makes it difficult to generalize the findings to different contexts. The results described have been extracted from research in a developing country, and it is likely that there are differences between countries.

### Conclusion

Students have a variety of health issues and have an increased demand for health information [36]. In the online era, the landscape of health information has changed, and the internet has increasingly become the main source of health information [52]. As Smith et al have pointed out, the question is no longer whether the internet can be an important source of information or not, but how its potential can be maximized [29].

Although students' access to online sources has increased substantially, they can only gain the most benefit from this information source by being able to effectively search for, evaluate, and use online information [29]. Moving forward, various stakeholders, including policymakers, information producers, health professionals, teachers, parents, and students themselves, should play their role well. Our study demonstrated the online health information–seeking behavior of a sample of female students in an Islamic developing country. Findings reported here have implications for communities with the same sociocultural status, although it can have lessons for other communities as well.

## Abbreviations

eHIQ: e-Health Impact Questionnaire
MCS: mental component summary
PCS: physical component summary
SF-36: 36-Item Short Form Health Survey
